# Nav channel binder containing a specific conjugation-site based on a low toxicity β-scorpion toxin

**DOI:** 10.1038/s41598-017-16426-x

**Published:** 2017-11-27

**Authors:** Tomoya Kubota, Bobo Dang, Joao L. Carvalho-de-Souza, Ana M. Correa, Francisco Bezanilla

**Affiliations:** 1 Department of Biochemistry and Molecular Biology, The University of Chicago, Chicago, IL 60637 USA; 2 Department of Chemistry, The University of Chicago, Chicago, IL 60637 USA; 3 Present Address: Department of Functional Diagnostic Science, Osaka University Graduate School of Medicine, Suita, Osaka, 5650871 Japan; 4 Present Address: Department of Pharmaceutical Chemistry, University of California, San Francisco, CA 94158 USA

## Abstract

Voltage-gated sodium (Nav) channels play a key role in generating action potentials which leads to physiological signaling in excitable cells. The availability of probes for functional studies of mammalian Nav is limited. Here, by introducing two amino acid substitutions into the beta scorpion toxin Ts1, we have chemically synthesized a novel binder [S14R, W50Pra]Ts1 for Nav with high affinity, low dissociation rate and reduced toxicity while retaining the capability of conjugating Ts1 with molecules of interests for different applications. Using the fluorescent-dye conjugate, [S14R, W50Pra(Bodipy)]Ts1, we confirmed its binding to Nav1.4 through Lanthanide-based Resonance Energy Transfer. Moreover, using the gold nanoparticle conjugate, [S14R, W50Pra(AuNP)]Ts1, we were able to optically stimulate dorsal root ganglia neurons and generate action potentials with visible light *via* the optocapacitive effect as previously reported. [S14R, W50Pra]Ts1 is a novel probe with great potential for wider applications in Nav-related neuroscience research.

## Introduction

Voltage-gated sodium (Nav) channels are the primary initiators of action potentials in excitable cells^1^. Mammalian Nav channels are complex membrane proteins composed by one pore forming α subunit and regulatory β subunits. The α subunit has four domains (DI-DIV), each of which contains six transmembrane segments (S1-S6). The S1-S4 form the voltage sensor domain (VSD), which plays a critical role in controlling dynamics of Nav channel conformation, and the S5-S6 segments of all domains forming one central pore^2–5^. Interestingly, it has been shown that Nav channels express not only on excitable cells but also on non-excitable cells including cancer cells and immune cells^6–8^. Therefore, development of Nav channel binders has been an important goal for use as potential research tools.

The most common specific binders to a target protein molecule used in research are antibodies^9^. Antibodies serve as powerful tools in biochemistry, immunohistochemistry, and imaging. Several antibodies for the Nav channel α subunits are commercially available, but most of them were generated using Nav channel cytosolic peptides as epitopes. Therefore, they have limited *in vivo* application potential for biophysical experiments or for neuroscience because the epitopes would not be accessible from the extracellular side. There are, however, very few reports of extracellularly targeted Nav antibodies, three of which are commercially available^10–13^.

Other specific binders for Nav channels are neurotoxins. Among these, beta scorpion toxins have been experimentally investigated using pharmacological and biophysical techniques as well as by computational modeling. The binding site in mammalian Nav channels has been identified to be formed by the S1-S2 linker and the S3-S4 linker of VSD in DII and the S5-S6 pore linker in DIII^14,15^. Functionally, all beta scorpion toxins shift the activation threshold of Nav channels in the direction of hyperpolarization. As described for Ts1, a beta scorpion toxin from *Tityus serrulatus* ^16^, as well as for Css4, a beta toxin purified from *Centruroides suffusus suffusus*, the mechanism of toxicity involves a “voltage-sensor trapping effect” whereby the toxin immobilizes the VSD of DII in an activated state, resulting in easier opening of Nav channels^17,18^. Additionally, it has been reported that a point mutation in Css4, E15R, dramatically reduces the “voltage-sensor trapping effect”, while still maintaining its binding affinity to Nav channels^19^.

We previously reported that the chemically synthesized Ts1 derivative, [W50Pra]Ts1 (called “Ts1-W50Pra” hereafter), showed the same potency as the natural toxin (IC_50_ values of 80 and 100 nM for TS1 and Ts1-W50Pra against the rat skeletal muscle Nav channels, Nav1.4, respectively)^20^. Chemical synthesis enabled us to incorporate an unnatural amino acid substitution, propargylglycine (Pra) for W50 (W50Pra) as a functionalizable site to conjugate with a variety of moieties for biological applications. Using Ts1-W50Pra conjugated to a fluorescent dye Bodipy (herein, Ts1-Bodipy), we have determined its binding location in rat skeletal muscle Nav channels (Nav1.4) using Lanthanide-based Resonance Energy Transfer (LRET), showing that this molecule is a powerful tool for biophysical research^21^. However, one of the concerns with Ts1-W50Pra is its strong toxicity, revealed as a change in threshold for channel opening, which may be detrimental for a living system.

In the study reported here, we have generated a novel potent Nav binder based on Ts1-W50Pra with significantly reduced toxicity by introducing a single amino acid substitution S14R, corresponding to E15R in Css4 toxin, [S14R, W50Pra]Ts1 (hereafter, “Ts1-S14R”). Using Ts1-S14R conjugated with Bodipy, [S14R, W50Pra(Bodipy)]Ts1 (herein, “Ts1-S14R-Bodipy”), LRET measurements showed that it binds to Nav1.4 at virtually the same location as Ts1-Bodipy. In addition, LRET data showed that, once Ts1-S14R-Bodipy binds to Nav1.4, it stays bound for at least 40 minutes in toxin-free solution, indicating that it can be used as a stable binder for Nav1.4. Moreover, we generated Ts1-S14R conjugated with gold nanoparticles, [S14R, W50Pra(AuNP)]Ts1 (called “Ts1-S14R-AuNP” hereafter) and we demonstrated that this conjugate can induce action potentials in rat dorsal root ganglia (DRG) neurons by visible light stimulation *via* the optocapacitive effect, as shown previously for Ts1-AuNP^22^. Collectively, Ts1-S14R can be a powerful probe with great potential for wider applications in Nav-related research for biophysics, pharmacology and neuroscience research.

## Results

### Total chemical synthesis of Ts1-S14R

**Figure 1:**
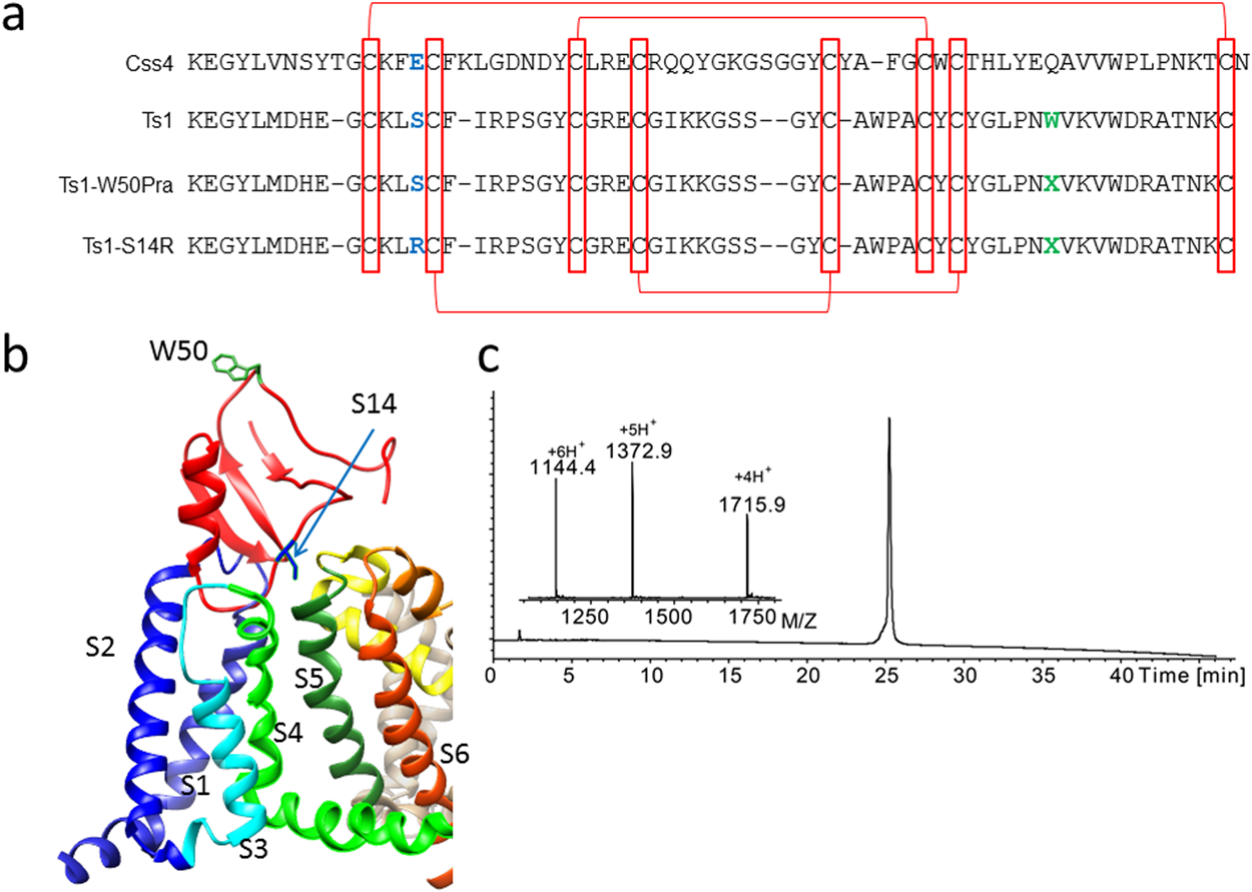
Synthetic Ts1-derivatives. (**a**) Sequence alignment of Css4, Ts1, and our synthetic Ts1-derivatives: Ts1-W50Pra and Ts1-S14R. Red rectangles and red lines indicate conserved cysteine residues which form disulfide bonds. S14 in Ts1 is the position where we introduced the arginine substitution as highlighted in blue, and W50 in Ts1 is the position where we introduced the L-propargylglycine (Pra: X) substitution highlighted in green. (**b**) Schematic representation of the Ts1 toxin interaction with the Nav channel. The structure of Ts1 cited from the Protein Data Base (PBD) #1NPI. Here, we used one domain of NavAb, a prokaryotic Nav channel from *Arcobacter butzleri* cited from PDB #4EKW as the structure of Nav channel. Drawing by the authors using a graphical picture obtained from PDB, based on the model proposed by Zhang, J. Z. *et al*.^14,15^ (not generated by molecular dynamic simulation). (**c**) Analytical LCMS data for Ts1-S14R; Calc. 6859.9 Da (Average isotope), Obsd. 6859.8 ± 0.2 Da.

In designing a Ts1 based binder with significantly reduced toxicity, we compared the primary sequences of Css4 and Ts1 as shown in Fig. 1a. Ts1 toxin has a similar sequence to Css4 (sequence similarity is 41.5%) and the cysteine residues which form important disulfide bonds for the folded structure are conserved between them. The point mutation E15R in Css4 has been shown to reduce its toxicity^19^. According to the sequence alignment, the corresponding point mutation in Ts1 would be S14R (Fig. 1b). We chemically synthesized Ts1-S14R by a similar strategy as Ts1-W50Pra (See also Material & Methods)^20^. Ts1-S14R showed good folding and purity in LCMS analysis (Fig. 1c).

### Pharmacological analysis using wild-type Nav1.4 in the presence of Ts1-W50Pra or Ts1-S14R

**Figure 2:**
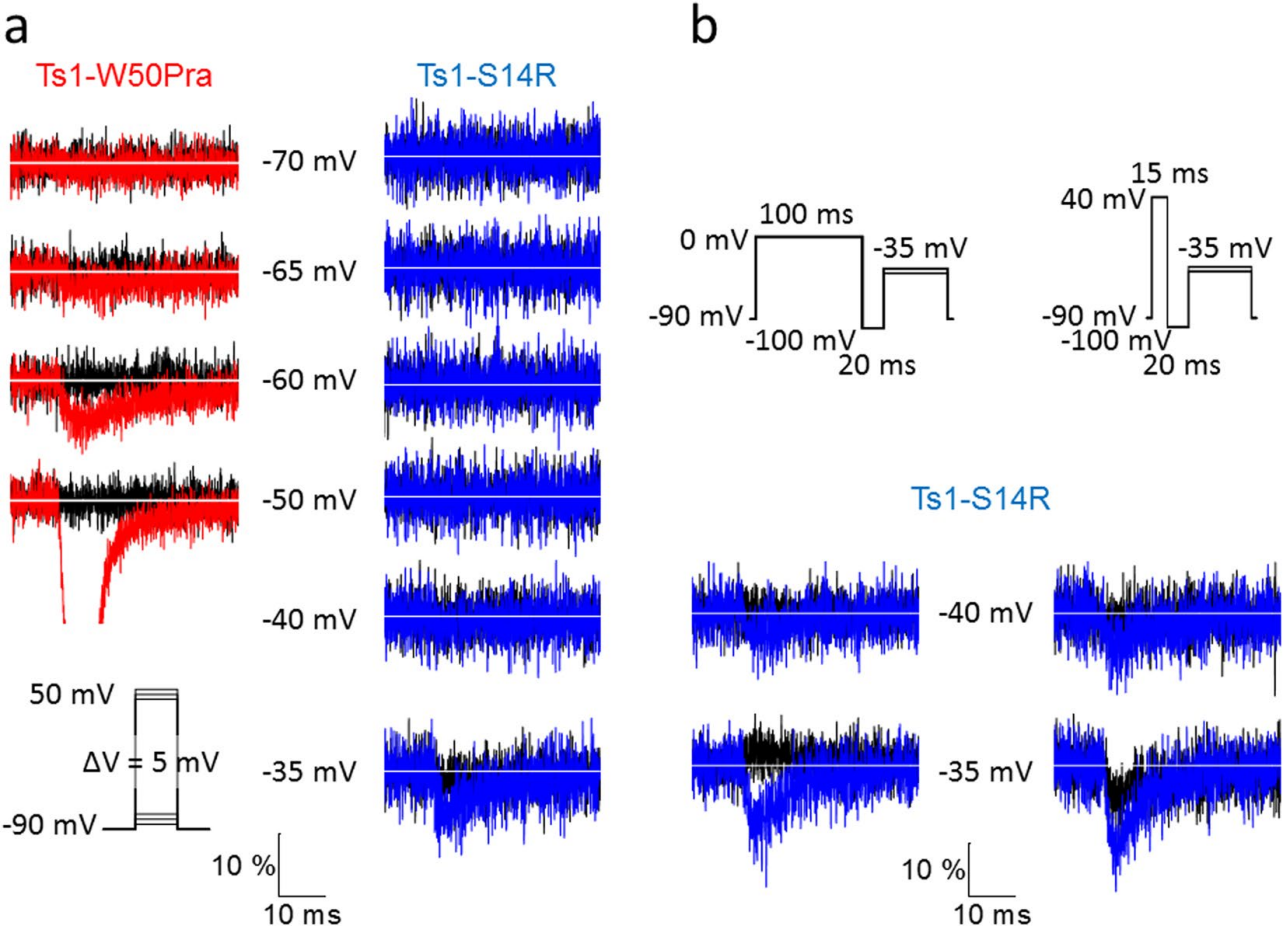
Voltage dependence of the ionic currents near threshold in wild-type Nav1.4 and in presence of Ts1-W50Pra or Ts1-S14R. (**a**) Ionic currents before applying toxin (black traces) and in the presence of toxin (Ts1-W50Pra in red and Ts1-S14R in blue). The traces in each column were obtained from the same cell before and after applying 1 µM of toxin and were superimposed after normalization by peak current. The inset in the lower-left part shows the protocol used. Scale bars are amplitudes (10% of peak current) and time (10 ms). (**b**) Currents before toxin (black traces) and in presence of Ts1-S14R when depolarized conditioning prepulse protocols were applied. These data was obtained from the same cell shown on the right column in (**a**). The protocols used are shown at the top. Current traces were obtained from the same oocyte. Scale bars, as in part a.

Next, we examined the pharmacological properties of Ts1-S14R by comparing it with Ts1-W50Pra using the cut-open oocyte voltage clamp technique (COVC) in *Xenopus* oocytes expressing Nav1.4^23^. First, we used an activation protocol with −90 mV as a holding potential as shown in Fig. 2a, inset. The contrast between the no toxin control (black) and the currents after applying 1 µM of Ts1-W50Pra to the bath (red) is exemplified by the superimposed traces in Fig. 2a, left column. While Na^+^ currents were already being detected with pulses to −65 mV (red traces) when in the presence of Ts1-W50Pra, Na^+^ currents in the absence of toxins (black traces) were only detected starting at pulses to −35 or −40 mV (black traces, right column). This result confirms that the threshold of Nav1.4 activation is shifted in the presence of Ts1-W50Pra. Conversely, after applying 1 µM of Ts1-S14R, Na^+^ currents appeared with pulses to −35 mV or −40 mV, not different to the toxin-free control, although the elicited current amplitude was larger in the presence of Ts1-S14R (Fig. 2a, right column). This result indicates that the “voltage sensor trapping effect” induced by the presence of Ts1-S14R was small.

The Css4 toxin with the E15R mutation showed low toxicity when a depolarizing conditioning prepulse was applied^19^. While Ts1 does not require a pre-depolarization to exhibit its effect, in order to examine the pharmacological effect of Ts1-S14R more precisely, we applied two different depolarized conditioning prepulse protocols as shown in the inset of Fig. 2b. The recordings shown were obtained in the absence (black traces) or presence of Ts1-S14R (blue traces) and at activation threshold voltages, −40 and −35 mV. The current recordings shown were obtained with the protocol illustrated immediately above each set of traces. In both protocols, the membrane was held at −90 mV, the test pulse was identical and it was preceded by a 20 ms, −100 mV repolarizing period. One protocol had a 100 ms depolarized conditioning prepulse to 0 mV (Fig. 2b, left side), while the other protocol gave a shorter, 15 ms, preconditioning pulse to a more depolarized voltage, 40 mV (Fig. 2b, right side). Using these two protocols, we measured Na^+^ ionic currents from the same cell shown in the right column of Fig. 2a. In both cases, in the presence of Ts1-S14R, Na^+^ ionic currents were detected with a −40 mV test pulse more clearly than with the no-prepulse activation protocol shown in Fig. 2a, indicating that Ts1-S14R retains some toxic effect but drastically reduced.

### Pharmacological analysis using wild-type Nav1.4 after pretreatment with Ts1-S14R

**Figure 3:**
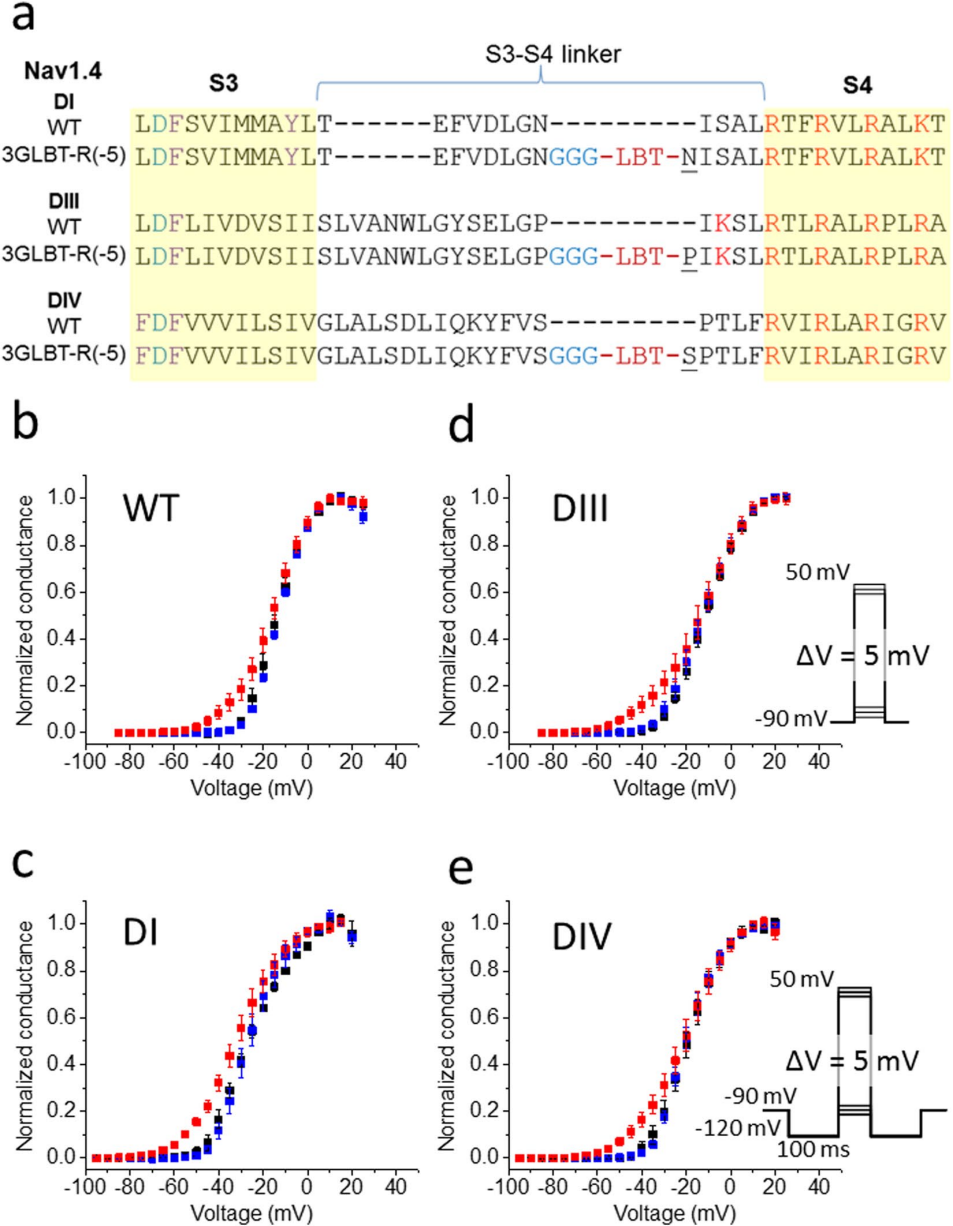
Voltage dependence of the conductance in wild-type Nav and Nav-LBTs after pretreatment with Ts1-W50Pra or Ts1-S14R. (**a**) Sequence alignment of the S3-S4 linkers of wild-type Nav1.4 in DI, DIII and DIV, compared to the respective S3-S4 linkers of the LBT constructs: DI-3GLBT-R(-5), DIII-3GLBT-R(-5) and DIV-3GLBT-R(-5), (adopted from Kubota T. *et al*.^21^). The underlined amino acids are overlapping amino acids with those prior to LBT insertions. (**b**–**e**) Normalized conductance of wild- type Nav channel (WT in **b**), DI-3GLBT-R(-5) (DI in **c**), DIII-3GLBT-R(-5) (DIII in **d**), and DIV-3GLBT-R(-5) (DIV in **e**). In each plot, blue squares are data obtained after pretreatment with 1 µM Ts1-S14R (WT: n = 5; DI: n = 4; DIII: n = 4; and, DIV: n = 5). Data plots before treatment (black squares) and after pretreatment with 1 µM Ts1-W50Pra (red squares) are adapted from Kubota T *et al*.^21^ (Black squares: WT, n = 5; DI, n = 4; DIII, n = 5; and, DIV, n = 5. Red squares; WT, n = 4; DI, n = 4; DIII, n = 4; and, DIV, n = 4). Error-bars are standard error of mean (SEM). The inset in (**d**) indicates the pulse protocol used for (**b**,**d**), and the inset in (**e**), that for (**c**,**e**).

As previously reported, once Ts1-W50Pra binds to Nav1.4, it can still display the “voltage-sensor trapping effect” even in toxin-free solution^21^. Briefly, we pretreated oocytes expressing Nav1.4 with 1 µM Ts1-W50Pra for 15 minutes in a glass dish. After washing them with toxin-free recording solution, we measured Na^+^ currents from Nav1.4 in the same toxin-free solution. As a result, Nav1.4 channels pretreated with 1 µM Ts1-W50Pra showed an activation threshold shifted by 15 mV in the direction of hyperpolarization (red plots in Fig. 3b adopted from Kubota T *et al*.^21^). Using a similar approach to test Ts1-S14R, we found that Nav1.4 pretreated with 1 µM of Ts1-S14R did not show a significant change in its threshold of activation (blue plots in Fig. 3b).

### LRET measurements of Nav-LBTs using Ts1-S14R-Bodipy

In interpreting the result obtained from oocytes pretreated with Ts1-S14R, i.e., a diminished effect, we could not exclude the possibility that Ts1-S14R did not bind to Nav1.4. To address this issue, we used the Lanthanide-based Resonance Energy Transfer (LRET) technique as reported before^21^ to confirm the binding of TS1-S14R to Nav1.4. Previously, we measured the intramolecular distance between Ts1-Bodipy and S4 in DI, DIII and DIV, respectively, using three different LRET donor clones (Nav-LBTs) and Ts1-Bodipy as acceptor^21^.

First, we confirmed that Ts1-S14R showed little or no effect on Nav-LBTs after pretreatment (blue plots in Fig. 3c–e). This observation in Nav-LBTs is consistent with that in wild-type Nav1.4 (Fig. 3b).

**Figure 4:**
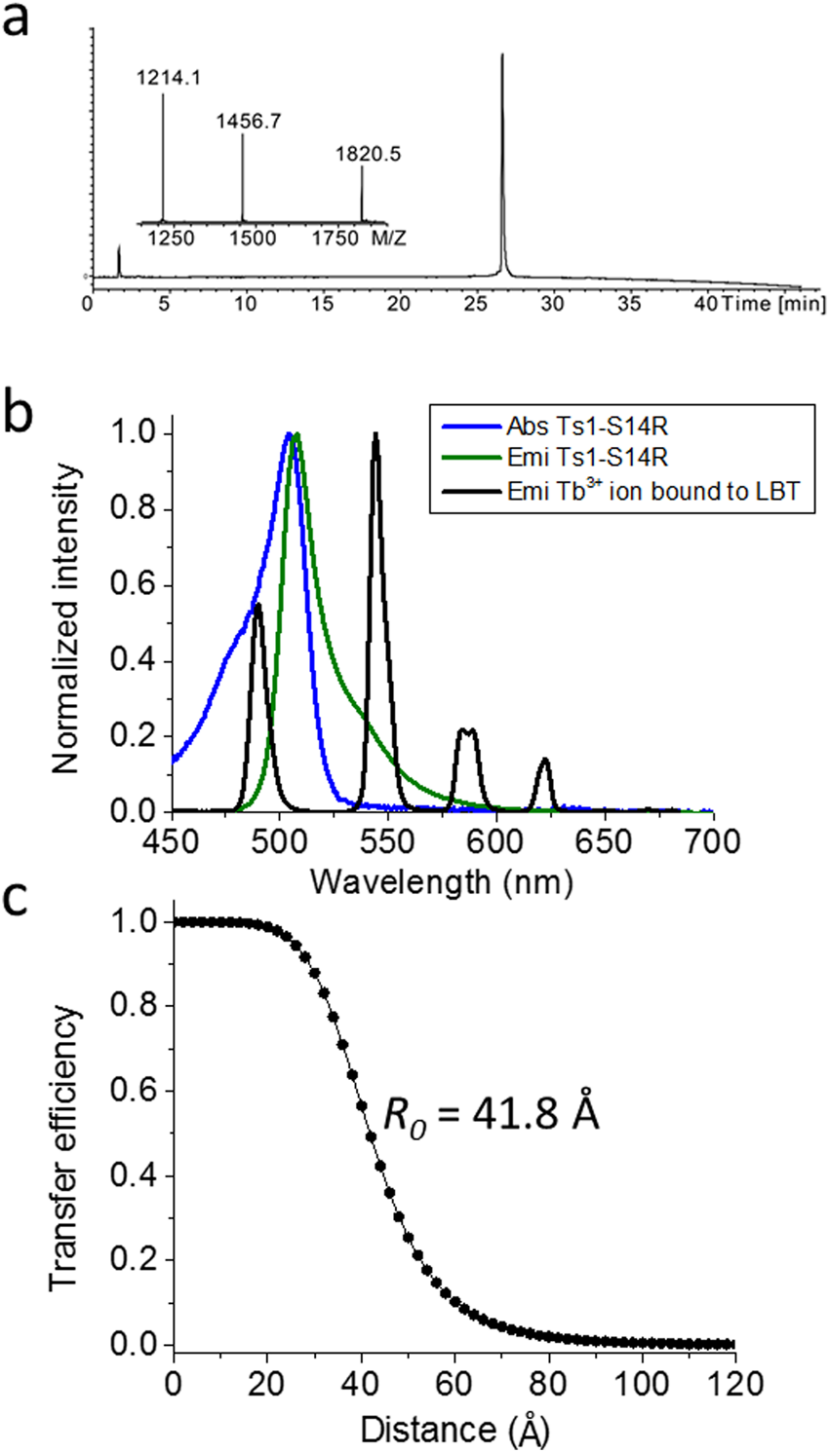
Calculation of R_0_, a ruler for LRET experiments, in the pair of Tb^3+^ ion bound to LBT and Ts1-S14R-Bodipy. (**a**) Analytical LCMS data for Ts1-S14R-Bodipy; Calc. 7278.1 Da (average isotopes), Obsd. 7278.4 ± 0.3 Da. (**b**) Overlap between the emission spectrum of Tb^3+^ bound to LBT (black solid line) and the absorption spectra of Ts1-S14R-Bodipy (blue solid line). The emission spectrum of Ts1-S14R-Bodipy is also shown (green solid line). (**c**) Energy transfer efficiency as a function of distance.

For LRET acceptors, we generated Ts1-S14R conjugated with Bodipy (Ts1-S14R-Bodipy) and the analytical LCMS data showed good folding and purity (Fig. 4a). We characterized the optical properties of Ts1-S14R-Bodipy and estimated an *R*_0_ of 41.8 Å using the Tb^+3^ in the LBT as donor (Fig. 4b,c).

**Figure 5:**
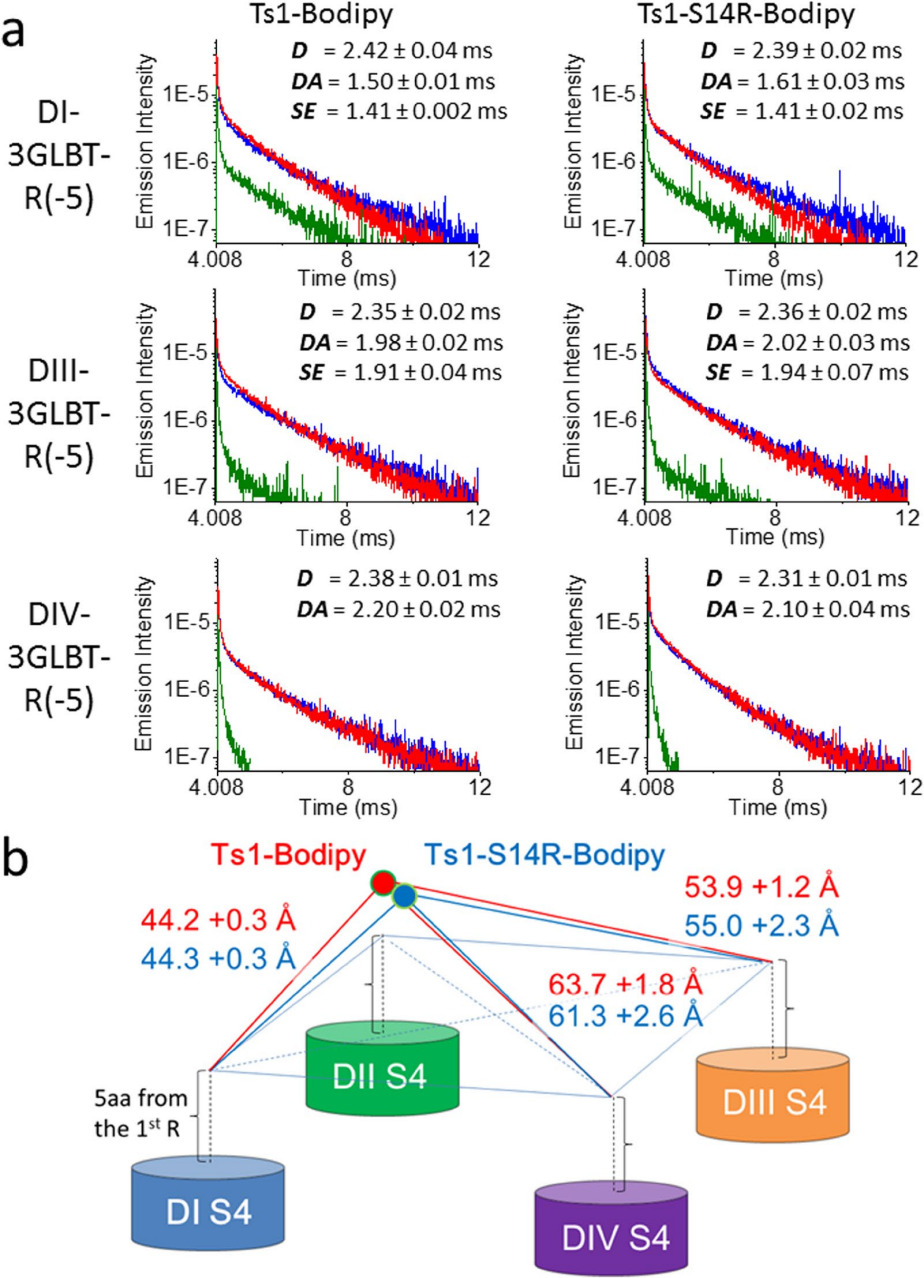
Distance measurements between Nav-LBTs and Ts1-Bodipy or Ts1-S14R-Bodipy by LRET. (**a**) Representative raw traces of donor signal (***D***: blue), donor in presence of acceptor signal (***DA***: red) and sensitized emission signal (***SE***: green) of Nav-LBTs and Ts1-Bodipy (left column, DI-3GLBT-R(-5): n = 5; DIII-3GLBT-R(-5): n = 6; and, DIV-3GLBT-R(-5): n = 4; adapted from Kubota T. *et al*.^21^), or Ts1-S14R-Bodipy (right column, DI-3GLBT-R(-5): n = 8; DIII-3GLBT-R(-5): n = 6: and, DIV-3GLBT-R(-5): n = 5). Time constants are shown as average values ± SEM. (**b**) Schematic structural map of the location of Ts1-Bodipy (red circle) and Ts1-S14R-Bodipy (blue circle) in Nav1.4, based on LRET measurements. Distance values of Ts1-Bodipy were adopted from Kubota T. *et al*.^21^. Distances are average values ± SEM calculated from the time constants.

We conducted LRET experiments using Ts1-S14R-Bodipy in the same way as was done previously with Ts1-Bodipy^21^. We used the same three different LRET donor clones; DI-3GLBT-R(-5), DIII-3GLBT-R(-5) and DIV-3GLBT-R(-5) (Fig. 3a). Here, the roman numbers (I, III, IV) indicate the Nav1.4 domain where the LBT was inserted; “3G” are the triple sequential glycines inserted at the N-terminal side of the LBT; and, “(-5)” indicates that the LBT is inserted five amino acids away from the first charge arginine of S4. The representative LRET signals including the donor ***D*** signal (blue trace), donor in presence of acceptor ***DA*** signals (red traces) and sensitized emission ***SE*** signals (green traces) are shown in Fig. 5. Using the time constants of signal decay in ***D*** and ***SE*** signals, the distance found between DI-3GLBT-R(-5) and Ts1-S14R-Bodipy was 44.3 ± 0.3 Å. Similarly, the distance between DIII-3GLBT-R(-5) and Ts1-S14R-Bodipy was 55.0 ± 2.3 Å. Construct DIV-3GLBT-R(-5) did not show large enough ***SE*** signals to obtain a reliable time constant. Therefore, the distance between DIV-3GLBT-R(-5) and Ts1-S14R-Bodipy was calculated using ***DA*** signals, resulting in 61.3 ± 2.6 Å. Taken together with the results from Ts1-Bodipy previously reported, these results confirmed not only that Ts1-S14R-Bodipy binds to Nav-LBTs but also that it shares virtually the same binding location as Ts1-Bodipy (Fig. 5b).

### Binding property of Ts1-S14R-Bodipy to Nav1.4 in LRET

**Figure 6:**
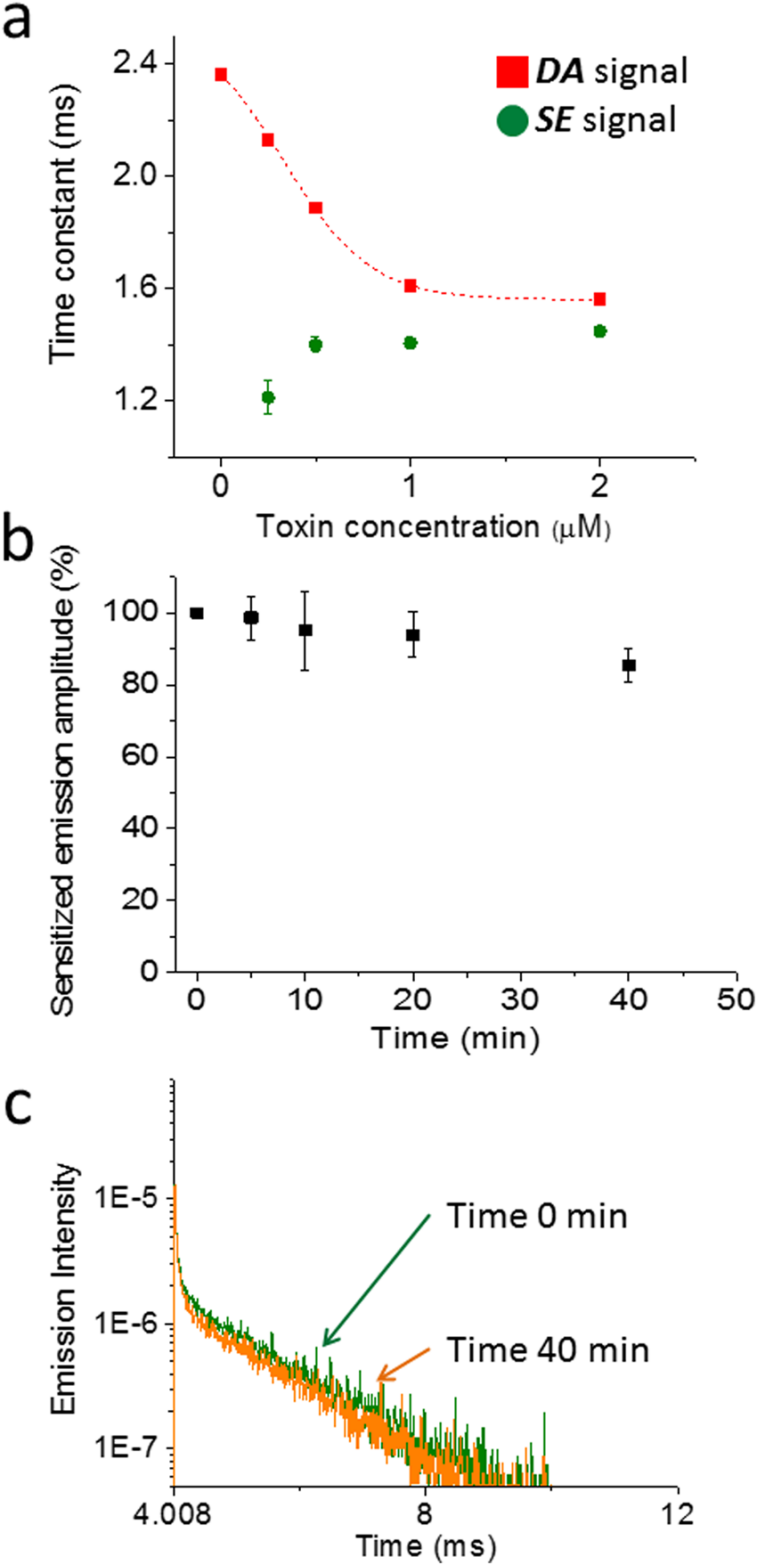
Binding stability of Ts1-S14R-Bodipy characterized by LRET using DI-3GLBT-R(-5). (**a**) Time constant of ***DA*** signals (red squares) are accelerated as the concentration of Ts1-S14R-Bodipy is increased, whereas that of ***SE*** signals (green circles) show a fixed value, around 1.4 ms (0 µM and 0.25 µM: n = 8; 0.5 µM: n = 6; 1 µM: n = 6; and, 2 µM: n = 4). The apparent dose-response curve of ***DA*** signals indicate that the proportion of Ts1-S14R-Bodipy binding to DI-3GLBT-R(-5) reach 50% at around 0.5 µM and reach 100% at around 1 µM. (**b**) The dissociation rate of Ts1-S14R-Bodipy as a function of time in toxin-free solution is very slow. We evaluated the binding fraction of Ts1-S14R-Bodipy by the amplitude of ***SE*** signals (n = 8). At 40 minutes after pretreatment by Ts1-S14R-Bodipy, 85% of the molecules are still bound. (**c**) Representative raw traces of ***SE*** signal at Time 0 min (green) and at Time 40 min (orange) in B.

To characterize the potential of Ts1-S14R as a binder to Nav1.4, we examined the binding potency of Ts1-S14R-Bodipy through LRET using DI-3GLBT-R(-5). We first obtained LRET signals after pretreating Nav-LBT expressing oocytes with varying concentrations of Ts1-S14R-Bodipy (Fig. 6a). At 0.25 µM, we could not get large enough sensitized emission signals to obtain a reliable time constant. However, at more than 0.5 µM, robust ***SE*** signals were obtained that consistently showed a time constant of 1.4 ms (green dots in Fig. 6a). The time constants of ***DA*** signals were accelerated by increasing the concentration of Ts1-S14R-Bodipy and the effect saturates at around 1 µM (red squares in Fig. 6a). This result indicates that approximately 0.5 µM of Ts1-S14R-Bodipy can bind to 50% of available Nav1.4. Next, we examined how long the Ts1-S14R-Bodipy stayed bound to DI-3GLBT-R(-5). Using the oocytes expressing DI-3GLBT-R(-5), we tracked the amplitudes of ***SE*** signals in toxin-free recording solution from “Time 0 min” after pretreatment with 1 µM of Ts1-S14R-Bodipy until 40 minutes after the pretreatment (“Time 40 min”). At 40 minutes after pretreatment, 85% of ***SE*** emission remained (Fig. 6b,c). This result indicates that the dissociation rate of Ts1-S14R-Bodipy is quite slow.

### Optocapacitive effect of gold nanoparticles conjugated to Ts1-S14R elicit action potentials in rat DRG neuron

**Figure 7:**
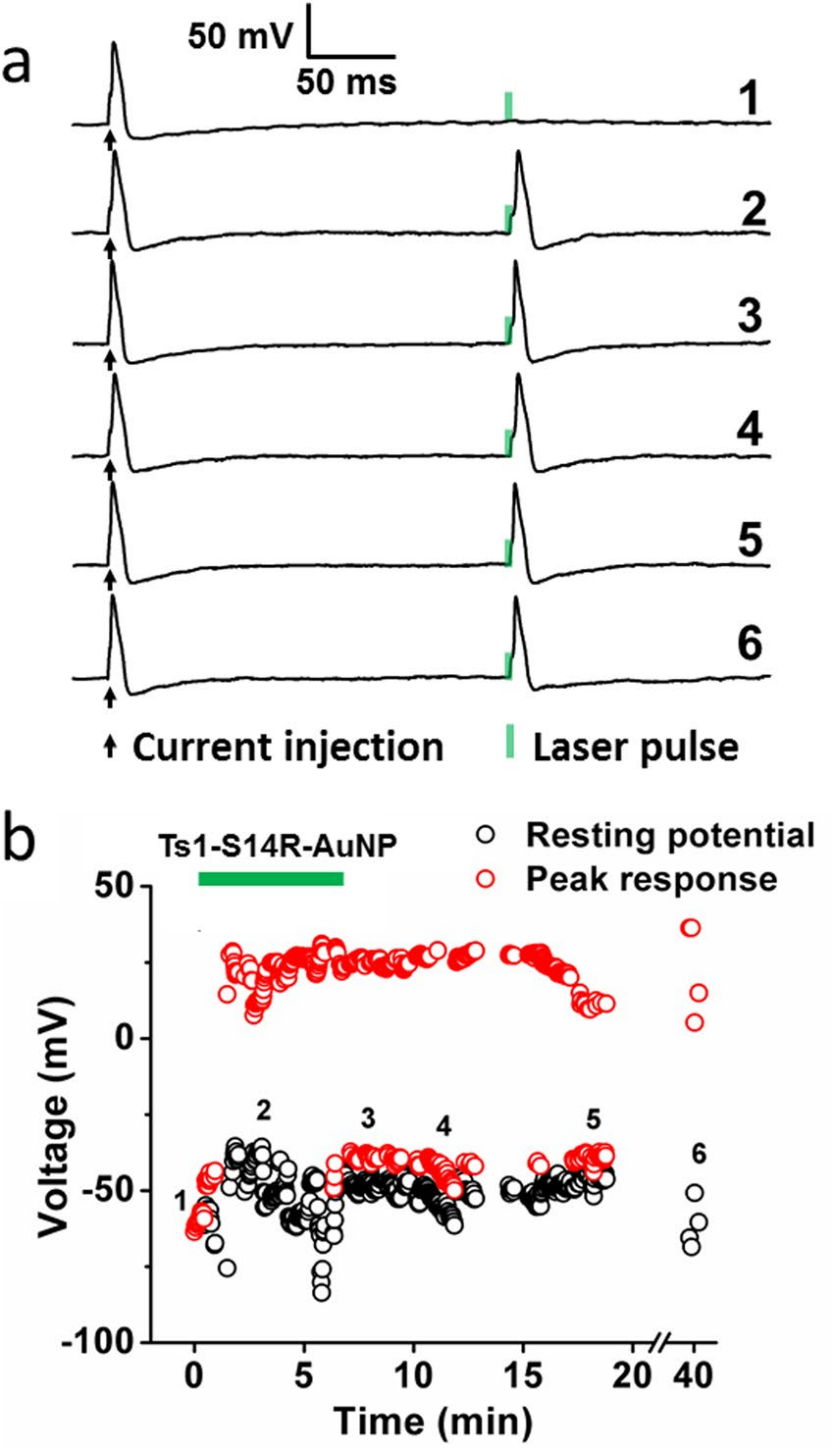
Ts1-S14R-AuNP provides time-stable generation of action potentials by photostimulation in isolated neurons. (**a**) Voltage recordings from a typical current clamp experiment in a DRG neuron showing action potentials elicited by a 800 pA current injection, followed by action potentials elicited by a 100 mW, 1 ms 532 nm laser pulse only after AuNPs were presented. Trace numbers denote a representative time point identified in part b. (**b**) Time course of the photosensitivity provided by Ts1-S14R-AuNP, showing resting potential values and peak response to the laser pulse over time. The green bar denotes the period of perfusion with Ts1-S14R-AuNP.

Taking advantage of the stable binding of Ts1-S14R to Nav channels, we generated a Ts1-S14R conjugate with gold nanoparticles (Ts1-S14R-AuNP) to activate neurons through the optocapacitive effect as previously described^22^. Briefly, AuNPs show plasmonic absorption of visible light, and the energy is emitted as heat. When placed close to a cell membrane, AuNPs can serve as light-to-heat transducers that can change the membrane temperature using low energy light. It is known that the membrane capacitance changes with temperature. By using AuNPs, one can quickly change the membrane capacitance, C, by a fast change in temperature, generating a depolarizing current proportional to V·dC/dt, where V is the membrane potential and t is time. With this technique, which we call “optocapacitance”, it is possible to make a cell light-sensitive without genetic modification, just by labelling it with AuNPs using a stable binder, in this case, Ts1-S14R. In neurons, depolarizations can be used to trigger action potentials, in a light pulse-by-light pulse fashion. We applied this technique to isolated dorsal root ganglia (DRG) neurons under current clamp by using the whole cell patch clamp technique. Every 5 seconds a depolarizing current injection pulse followed by a 100 mW, 1 millisecond, 532 nm light pulse were applied to the DRG neuron being tested. Current pulses were used to probe the cell excitability. As expected, in the absence of Ts1-S14R-AuNP, only current pulses are able to reliably trigger action potentials while light pulses cannot (Fig. 7, #1). A few minutes after these recordings were made, 20 nM Ts1-S14R-AuNP was applied to the neuron by a localized perfusion system (see Material & Methods), and an action potential was triggered by the light pulse in addition to the current pulse (Fig. 7, #2). Remarkably, even after washout from the neuron of the unbound Ts1-S14R-AuNP, the light pulse could trigger action potentials for up to 40 minutes, being limited by the duration of the patch clamp experiment (Fig. 7, #3–#6). These data suggest the successful steady attachment of the Ts1-S14R-AuNP to the neurons, consistent with the observation in the LRET experiment (Fig. 6).

## Discussion

In this study, we generated a novel Nav binder (Ts1-S14R) with high affinity and extremely low dissociation rate. The great advantage of this binder is that we can conjugate different molecules of interest for a variety of physiological studies. Because of its minimal toxicity, this can be a probe with great potential for wider applications in Nav channel related research including structural, pharmacological and neuroscience studies.

As stated before, several antibodies for the Nav channel α subunit are commercially available, but most of them are generated using cytosolic peptides as epitopes, mainly in the linkers between DI-DII, DII-DIII, and DIII-DIV or in the C-terminal. To our knowledge, there are only three commercially available and one literature reported antibodies for Nav channels whose epitopes are in the extracellular face of the channel^10–13^.

**Figure 8:**
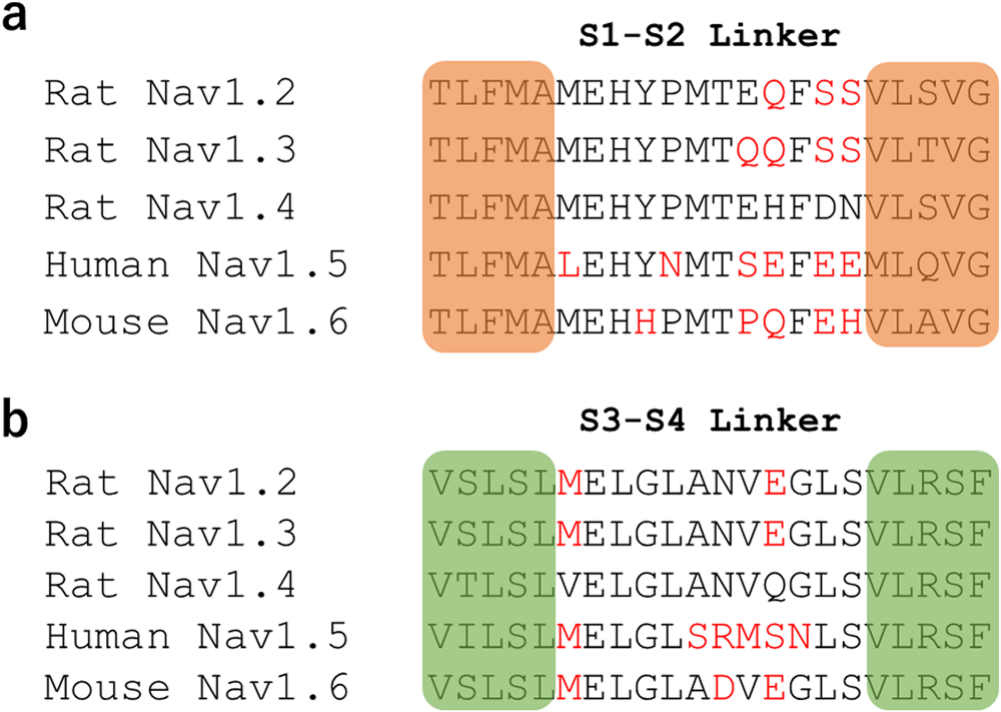
Alignment of Nav amino acid sequences proposed to contain the mammalian β-scorpion binding site. Alignment of amino acid sequences of DII S1-S2 linker (**a**) and S3-S4 linker (**b**) of rat Nav1.2, rat Nav1.3, rat Nav1.4, human Nav1.5 and mouse Nav1.6, area of the Nav protein in which Ts1-S14R is expected to bind. Amino acids that differ from the rat Nav1.4 sequence are highlighted in red.

Comparing with extracellular targeting antibodies, our Ts1-S14R has several advantages. Firstly, Ts1-S14R has great potential for *in vivo* imaging. As Ts1-S14R recognizes the three dimensional structure composed of the DII-VSD and DIII-pore loop, it is advantageous to use Ts1-S14R to target functional Nav *in vivo* for imaging as compared to antibodies since the available epitopes for antibodies are not well exposed to the extracellular medium. Secondly, Ts1-S14R has high accessibility to the target because of its small size. Ts1-S14R is quite small (7 kDa) compared to antibodies (~150 kDa) and this is a big advantage for biophysical experiments. Thirdly, we can easily achieve site specific conjugation of any moiety of interest to Ts1-S14R. The conjugation site located in Ts1 is facing up to the extracellular side such that the binding affinity is minimally affected, as we have demonstrated. Fourthly, Ts1-S14R is much less toxic for Nav1.4. We therefore do not need to be concerned by the side effects that can be caused by the toxicity of wild-type Ts1. Finally, the binding affinity of Ts1-S14R is almost equivalent to that of usual antibodies. In LRET experiments, Ts1-S14R-Bodipy showed 50% binding at around 0.5 µM and kept bound for 40 minutes. Since LRET is an uncommon method to evaluate the binding affinity, we cannot do a direct comparison of the affinity of Ts1-S14R to that of antibodies. However, LRET data suggests that their binding affinities are comparable. A disadvantage of Ts1-S14R is that the binding specificity of Ts1 on different Nav subtypes might be low. A recent publication showed pharmacological effects on each Nav subtype and demonstrated that Ts1 binds to rat Nav1.2, rat Nav1.3, rat Nav1.4, human Nav1.5 and mouse Nav1.6 channels^24^. This previous study showed that Ts1 made four of five subtypes activate easier at 100 nM although the extent of the toxic effect on each Nav subtype was different. For human Nav1.5, Ts1 caused marked reduction of ionic currents without a shift of activation threshold, which indicated that the binding mode of Ts1 against human Nav1.5 may be different from that against other 4 subtypes. As our study included only Nav1.4, we have no experimental evidence to address whether or not S14R in Ts1 has reduced toxicity against other Nav subtypes. However, considering that the amino acid sequences of the linkers between S1-S2 and S3-S4 in the Domain II of rat Nav1.2, rat Nav1.3, rat Nav1.4, human Nav1.5 and mouse Nav1.6, although not identical, are quite similar (Fig. 8), it could be expected that the effect of Ts1-S14R on all these other Nav subtypes would be similar to that on rat Nav1.4. We anticipate that Ts1-S14R will be useful as an anti-Nav binding protein against skeletal muscle (Nav1.4), that may be also used against Nav expressed in neurons (Nav1.2, Nav1.3 and Nav1.6) and, possibly, in the heart (Nav1.5).

## Conclusion

In the work reported here, we describe Ts1-S14R as a substantially non-toxic binder for Nav channels; its low toxicity spares us from concerns of disturbing the biological systems or even causing damage as when using the wild-type toxin. Ts1-S14R has high affinity for Nav channels and is resistant to washing with toxin-free buffer. Ts1-S14R can be site-specifically conjugated to molecules of interest, suggesting that this is a novel probe with great potential for a wide number of applications. Because there are very limited number of probes available for studying Nav channels, we think that Ts1-S14R provides a valuable tool for Nav-related research.

## Methods

### Total chemical synthesis of Ts1 derivatives

#### Reagents

Biotin-PEG3-Azide was purchased from Sigma-Aldrich. 20 nm AuNP-Streptavidin was obtained from Nanopartz. Slide-A-Lyzer MINI Dialysis Devices 20 K MW cut-off was obtained from Thermo Scientific. Other chemicals were purchased from Sigma-Aldrich.

#### Synthesis of [S14R,W50Pra]Ts1. CONH_2_ (Ts1-S14R)

Ts1-S14R was prepared in a similar fashion as previously described^20^ except for the folding reaction. In the folding reaction, Ts1-S14R polypeptide (6 mg, 0.85 μmol) was first dissolved in 100 mL DMSO, folding buffer (0.5 M L-Arg·HCl, 100 mM Tris, 1 mM cystine·2HCl, 8 mM cysteine, pH 8.5) 400 mL was then added to DMSO at ice water bath, reaction was left overnight at 4 °C. Folded product was first extracted out by doing solid phase extraction and then purified using semi-prep column to give 1.8 mg (0.26 μmol, 30.6%) product.

#### Labeling Ts1-S14R with BODIPY® FL-γ-azido-2-Aminobutanoic acid (Abu). (Ts1-S14R-Bodipy)

BODIPY® FL-γ-azido-Abu was prepared by adding BODIPY® FL succinimidyl ester (2.5 mg, 6.4 μmol in 1.25 mL DMF) and γ-azido-2-Aminobutanoic acid (Abu) (11.2 mg, 77.8 μmol) to 12 mL 0.1 M NaHCO_3_; the reaction was left for 20 minutes. Product was purified by solid phase extraction using Alltech C18 large pore cartridge. In the labeling reaction, to 2.0 mL of degassed buffer (1 M guanidine hydrochloride, 200 mM Tris, 40 mM TCEP hydrochloride) was added 80 μL 1 M CuSO_4_ to generate Cu(I) *in-situ*; the buffer was heated to fully consume TCEP hydrochloride. After cooling to room temperature, the pH was adjusted to 8.7. Ts1-S14R (0.5 mg, 72.8 nmol) was dissolved in 400 μL degassed water then BODIPY® FL-γ-azido-Abu (250 μg, 598 nM) and 400 μL click reaction buffer were added, the reaction was left for 15 minutes before HPLC purification to obtain product (0.37 mg, 50.8 nmol 69.8% yield. Observed mass 7278.4 ± 0.3 Da, calc. 7278.1 Da (av. isotopes)).

#### Conjugation of Ts1-S14R with Azide-PEG3-Biotin. (Ts1-S14R-PEG3-Biotin)

In a typical reaction, to 2.0 mL of degassed buffer (1 M Guanidine hydrochloride, 200 mM Tris, 40 mM TCEP hydrochloride) was added 80 μL 1 M CuSO_4_ to generate Cu(I) *in-situ*; the buffered solution was heated up to ensure the TCEP hydrochloride was fully consumed. After cooling to room temperature, the click reaction buffer pH was adjusted to 8.7 before use. Ts1-S14R (0.2 mg, 29.1 nmol) was dissolved in 200 μL degassed water then Azide-PEG3-Biotin (40 μL of 2.5 mg/mL in DMF) and 140 μL click reaction buffer were added to the solution. After 10 minutes, the reaction product was purified on a C4 analytical column (60 μg, 8.2 nmol, 28.2% yield. Obsd. 7303.6 ± 0.3 Da, Calc. 7303.9 Da (av. isotopes)). The amount of protein product was determined from the OD 280 nm measured on a NanoDrop spectrophotometer.

#### Conjugation of Ts1-S14R-PEG3-Biotin with 20 nm Streptavidin-AuNP (Ts1-S14R-AuNP)

Ts1-S14R-PEG3-Biotin (4 μM, 50 μL in 1X external solution) and 20 nm AuNP-Streptavidin (54 nM, 50 μL in 1X external solution) were mixed and shaken well. After 5 hours incubation at 4 °C, the mixture was transferred to the 20 K MW cut-off MINI dialysis tube and dialyzed against 700 mL 1X external solution 3 times at 4 °C (4 hours, overnight, 4 hours) with gently stirring to remove non-bound Ts1-S14R-PEG3-Biotin. This dialyzed AuNP-Streptavidin-Biotin-PEG3-Ts1-S14R conjugate was used directly for assays.

#### Reverse phase HPLC and LC-MS analysis

Analytical reversed phase HPLC and LC-MS were performed using an Agilent 1100 series HPLC system equipped with an online MSD ion trap. Column used was Phenomenex Aeris WIDEPORE 3.6 μm C4, 150 × 4.6 mm. Chromatographic separations were performed using a linear gradient of 5–45% acetonitrile (0.08% TFA) versus water (0.1% TFA) over 40 minutes with column temperature 40 °C. Flow rates were controlled at 0.9 mL/min. Peptide and protein detection was by UV absorption at 214 nm, and masses were obtained by online electrospray mass spectrometry.

#### Preparative HPLC

The product from the click reaction was purified using a Phenomenex Aeris WIDEPORE 3.6 μm C4, 150 × 4.6 mm column. A shallow gradient of acetonitrile (0.08% TFA) versus water (0.1% TFA) was used. Flow rates were controlled at 0.9 mL/min. Fractions were collected and those fractions containing the desired product, identified by analytical LC and mass spectrometry, then combined and lyophilized.

#### Preparation of Ts1 derivatives

All Ts1 derivatives were diluted in the external solution (see Electrophysiology) containing 0.5–1% Bovine Serum Albumin (BSA). The concentrations of Ts1-W50Pra and Ts1-S14R were obtained by spectrophotometer (Nanodrop, Thermo scientific, USA). The concentrations of Ts1-S14R-Bodipy was calculated based on the extinction co-efficient values and the peak values of their absorption spectra obtained by spectrophotometer (Cary 60 UV-Vis, Agilent Technologies, USA) (See also Extinction coefficient measurements in the recording solution).

#### Animals

For the heterologous expression of rat skeletal muscle voltage-gated sodium channel (Nav1.4), we used oocytes extracted from the ovaries of mature female *Xenopus laevis* frogs, with ovary lobes extracted *via* survival surgery under anesthesia. *Xenopus laevis* frogs were used just as tissue (oocytes) providers. For the optocapacitive stimulation experiments, rat dorsal root ganglia were obtained from P1-P3 Sprague-Dawley animals. All animal protocols in this study were approved by the University of Chicago Institutional Animal Care and Use Committee (IACUC). All experimental procedures were performed in accordance with the relevant guidelines and regulations of the Biosafety Committee of the University of Chicago.

#### Molecular biology and oocyte preparation

Clones and oocytes were prepared as previously described^21^. Briefly, the α subunit and β1 subunit of rat skeletal muscle voltage-gated sodium channel (Nav1.4) were cloned into pBSTA vector. The cRNA were transcribed *in vitro* using T7 mMESSAGE cRNA kit (Ambion) and injected in a molar ratio 1:1 into *Xenopus* oocytes. For the electrophysiological experiments, freshly isolated oocytes were injected with 1 ng of cRNA and kept in the Standard Oocytes Saline (SOS) solution: 96 mM NaCl, 2 mM KCl, 1 mM MgCl_2_, 1.8 mM CaCl_2_, 10 mM HEPES, 200 mg/L sodium pyruvate, pH 7.4, with 100 µg/ml gentamycin for 1–2 days at 18 °C. For LRET experiments, the oocytes were injected with 75 ng of cRNA and incubated in SOS after injection for 5–6 days at 12 °C, and after then incubated for 1–2 days at 18 °C.

#### Electrophysiology

The oocytes were mounted in the Cut-open oocyte voltage clamp (COVC) set-up. The external solution contained 57.5 mM n-methylglucamine (NMG)-methylsufonate (MS), 57.5 mM Na-MS, 10 mM HEPES, and 2 mM Ca-MS, pH 7.4. The internal solution contained 103.5 mM NMG-MS, 11.5 mM Na-MS, 10 mM HEPES, and 2 mM EGTA, pH 7.4. When Ts1 derivatives were dissolved in these solutions, we added 0.5–1% BSA. When recording ionic currents in the presence of Ts1 derivatives, the toxins were applied to both the upper and the guard chambers of COVC set-up. When the oocytes were pretreated with Ts1 derivatives for electrophysiology and LRET, 1 µM Ts1 derivative was applied for 15 minutes and then washed first in SOS solution and then in the recording solution before the measurement. Because Ts1-derivatives are very sticky to plastic materials, we used glass dishes during the whole process.

Ionic currents were recorded by digitizing them by a 16 bit A/D converter, as described previously^21^. Ionic currents were sampled at 10 μs/point. The data acquisition program was developed in-house. Linear leak and membrane capacitive currents were subtracted using a P/6 protocol from a subtracting holding potential of −100 mV. All data were obtained at 12–14 °C.

Conductance was calculated as G(V) = I_peak_(V)/(V − E_rev_), where the reversal potential, E_rev_ was measured experimentally for each oocyte. Statistical significance was determined using an unpaired t-test. Errors indicate standard error of means (SEM).

#### LRET recordings

LRET recording was done as described previously^21^. The donor is Tb^3+^ bound to the genetically encoded modified lanthanide-binding-tag motif (LBT), YIDTNNDGWYEGDELLA, which binds Tb^3+^ with high affinity (Kd = 57 nM). The modified LBT has an additional tryptophan, YWDTNNDGWYEGDELLA, to improve Tb^3+^ donor signal of Nav-LBT constructs. The optical setup for LRET measurements has been described previously^21,25–29^. The LRET recording solution contained 92 mM NMG-MS, 23 mM Na-MS, 10 mM HEPES, 2 mM Ca-MS and 5 µM Tb^3+^, pH 7.4. During the experimental process, each oocyte was placed in separated wells to be identified.

The emission spectrum of Tb^3+^ ions bound to LBT was measured in our laboratory. The absorption and emission spectra of Ts1-S14R-Bodipy were obtained with a spectrophotometer (Agilent Cary 60, Agilent Technologies). R_0_ for the pair of Tb^3+^ ion bound to LBT and Ts1-S14R-Bodipy was obtained as previously reported^21^. The emission spectra were obtained with a Fluorimeter (Photomultiplier Detection Systems, Photon Technology International).

### Optocapacitive stimulation experiments

#### Cell Culture Protocol

Rat dorsal root ganglia were excised from P1-P3 Sprague-Dawley animals following decapitation and were immediately placed in DMEM (Life Technologies, Grand Island, New York, Cat#: 21063-029) on ice. Ganglia were rinsed multiple times with EBSS (132 mM NaCl, 5.3 mM KCl, 10 mM HEPES, 1 mM NaH_2_PO_4_, 5.5 mM glucose, pH 7.4) then digested with 0.25% Trypsin (Worthington, Lakewood, New Jersey, Cat#: TRL3) in EBSS for 20 minutes at 37 °C under gentle shaking. After digestion, the material was centrifuged, the supernatant removed and EBSS + 10% FBS (Fetal bovine serum, ATCC, Manassas, Virginia, Cat#: 30-2020) was added. The digested ganglia were then mechanically triturated through Pasteur pipettes of decreasing size. A final centrifugation was done, supernatant removed and DMEM + 5% FBS was added. Cells were seeded into sterilized PLL (Poly-L-lysine solution, Sigma-Aldrich, St. Louis, Missouri, Cat#: P8920)-treated glass-bottomed culture dishes and allowed to sit for 30 minutes to facilitate DRG cell adhesion to the glass. Finally, the dishes were flooded with DMEM + 5% FBS + 100 U/ml penicillin (Sigma-Aldrich, Cat#: 13750) + 100 ug/ml streptomycin (Sigma-Aldrich, Cat#: S6501) and incubated at 37 °C with 5% CO_2_ until use.

#### Experimental setup

Cell dishes containing DRG cells and bath solution (132 mM NaCl, 4 mM KCl, 1.2 mM MgCl_2_, 1.8 mM CaCl_2_, 10 mM HEPES, 5.5 mM glucose, pH 7.4) were mounted on a Zeiss IM 35 microscope (Carl Zeiss Microscopy, Thornwood, New York) and visualized through objective lenses ranging from 10x/0.25NA to 40x/0.55NA. Patch pipettes were pulled on a Sutter Instruments P-2000 CO_2_ laser micropipette puller (Novata, California) and flame polished to produce approximately 2 MΩ resistances when filled with internal pipette solution (10 mM NaCl, 130 mM KF, 4.5 mM MgCl_2_, 10 mM HEPES, 9 mM EGTA, 2 mM ATP, pH 7.3). Voltage or current through cell membranes were clamped using an Axopatch 200B amplifier (Molecular Devices, Sunnyvale, California). An analog waveform from the data acquisition board (Innovative Integration SBC-6711-A4D4, Simi Valley, CA) drove the amplifier to clamp the current through the cell’s membrane. The amplifier membrane voltage output was digitized with the data acquisition board and stored in a personal computer for analysis. Ts1-S14R-AuNP were delivered *via* one side of a theta capillary tube and the other side was filled with bath solution for washing. Each side of the tube was connected to independently-controlled pressurized air. The Ts1-S14R-AuNP were stimulated with a 532 nm DPSS laser beam (UltraLasers, Ontario, Canada), modulated with an acousto-optic modulator (NEOS Technologies, Gooch & Housego, PLC., Melbourne, Florida) and delivered to the 40x objective.

#### Optocapacitance stimulation

DRG neurons were patched under voltage-clamp, with seal resistance monitored until the giga-seal was achieved. In whole-cell patch clamp configuration, the current clamp mode was activated and the cell excitability was tested with 1 ms current injection pulses of increasing amplitudes. The minimal current amplitude to trigger action potentials ranged from 300 pA to 700 pA for different cells. The membrane voltage was filtered at 5 kHz and digitized at 20 kHz. We utilized the method described previously to stimulate neuronal activity with light^22^. Briefly, after a DRG neuron under current clamp was tested for excitability by injecting depolarizing current, solution containing 20 nM Ts1-S14R-AuNP was perfused close to the cell and its photosensitivity to a 1 ms, 532 nm light pulse was monitored by observing its membrane voltage. When the depolarization effect induced by the light was enough to trigger an action potential, the perfusion continued for 5 minutes and then the solution with Ts1-S14R-AuNP was washed out.
